# Exploring Diet Quality between Urban and Rural Dwelling Women of Reproductive Age

**DOI:** 10.3390/nu9060586

**Published:** 2017-06-08

**Authors:** Julie C. Martin, Lisa J. Moran, Helena J. Teede, Sanjeeva Ranasinha, Catherine B. Lombard, Cheryce L. Harrison

**Affiliations:** 1 Monash Centre for Health Research and Implementation, School of Public Health and Preventative Medicine, Monash University, Melbourne 3004, Australia; Julie.C.Martin@monash.edu (J.C.M.); lisa.moran@monash.edu (L.J.M.); helena.teede@monash.edu (H.J.T.); sanjeeva.ranasinha@monash.edu (S.R.); 2 Endocrinology and Diabetes Units, Monash Health, Melbourne 3004, Australia; 3 Department of Nutrition and Dietetics, School of Public Health and Preventative Medicine, Monash University, Melbourne 3004, Australia; catherine.lombard@monash.edu

**Keywords:** dietary assessment, diet quality, nutrition, diet, rural-urban, women, reproduction, weight gain prevention

## Abstract

Health disparities, including weight gain and obesity exist between urban and rural dwelling women. The primary aim was to compare diet quality in urban and rural women of reproductive age, and secondary analyses of the difference in macronutrient and micronutrient intake in urban and rural women, and the predictors of diet quality. Diet quality was assessed in urban (*n* = 149) and rural (*n* = 394) women by a modified version of the Dietary Guideline Index (DGI) energy, macronutrient and micronutrient intake from a food frequency questionnaire (FFQ) and predictors of diet quality. Diet quality did not significantly differ between urban and rural women (mean ± standard deviation (SD), 84.8 ± 15.9 vs. 83.9 ± 16.5, *p* = 0.264). Rural women reported a significantly higher intake of protein, fat, saturated fat, monounsaturated fat, cholesterol and iron and a higher score in the meat and meat alternatives component of the diet quality tool in comparison to urban women. In all women, a higher diet quality was associated with higher annual household income (>$Australian dollar (AUD) 80,000 vs. <$AUD80,000 *p* = 0.013) and working status (working fulltime/part-time vs. unemployed *p* = 0.043). Total diet quality did not differ in urban and rural women; however, a higher macronutrient consumption pattern was potentially related to a higher lean meat intake in rural women. Women who are unemployed and on a lower income are an important target group for future dietary interventions aiming to improve diet quality.

## 1. Introduction

Women of reproductive age are a high-risk group for progression to obesity with higher annual weight gain compared to older women and men generally [1,2]. Weight gain in women is a complex interplay of genetic, environmental and social factors and in line with global consensus, prevention of weight gain is now an international priority [3]. As women are role models to their children [4], and the main food preparers for their family [5], targeting young women for weight gain prevention is urgently required and may yield benefits for the next generation [6,7,8,9,10].

In developed countries, obesity prevalence has been reported to be higher among rural women in comparison to urban women [11], with higher progressive background weight in rural women demonstrated longitudinally (rural 725 g/year; urban 606 g/year) [1]. Potentially contributing factors include higher rates of smoking and alcohol consumption, poorer diet, lower levels of physical activity, and higher levels of psychosocial stress [11]. Many of these health differentials are related to a higher level of socioeconomic disadvantage in rural populations [11,12], where access to health care and health promotion programs is reduced [13] and the cost of maintaining healthy behaviors including a healthy diet is higher [14].

Diet is one risk factor that can be modified to prevent weight gain, improve health and reduce overall disease risk [15]. Assessing diet quality within populations provides a holistic assessment of nutrient adequacy, compliance with dietary guidelines and provides insight to the impact of diet quality on health outcomes [16]. Higher diet quality is inversely related to all-cause mortality, cardiovascular risk, cancer risk and risk of type 2 diabetes adjusted for age, sex, body mass index (BMI), energy intake, education and physical activity [17].

Despite this, few studies have measured diet quality in younger, reproductive aged women and studies in higher risk settings including rural populations, compared to urban settings, are conflicting. For example, while some studies in Australia [18], Canada [19], Mexico [20] and Poland [21] report better diet quality in urban women, compared to their rural counterparts, other studies conducted in Greece and Africa report the contrary [22,23], while other studies reported insignificant differences [18,24]. Variations in data collection methodology, assessment of diet quality, urban and rural definitions and socioeconomic differences between developed and developing countries may, in part, account for the differences reported. However, as these studies are not specific to reproductive aged women, it is unknown if diet quality differs in younger women in urban and rural settings. The assessment of diet quality in settings and populations at high risk for weight gain may better inform the development of targeted weight gain prevention programs [25,26,27]. Therefore, we aimed to assess whether diet quality, as well as macronutrient and micronutrient intake differs between urban and rural women of reproductive age and explore predictors of diet quality recruited as part of a randomized controlled healthy lifestyle intervention.

## 2. Materials and Methods

### 2.1. Study Design

This study was a sub-study of the Healthy Lifestyles (HeLp-her) cluster randomized controlled trials (RCTs). HeLP-her was developed to prevent excessive weight gain in women of reproductive age by supporting a healthy lifestyle and promoting health and wellness through low intensity behavior change techniques [7,10,28]. Participants included those at baseline recruited as part of two separate cluster RCTs. The first trial was conducted in 2006 in urban Melbourne, Australia (HeLP-her urban) [6,7], followed by the second trial in 2012 in rural Victoria, Australia (HeLP-her rural) [8]. HeLP-her urban recruited women from one local government area in metropolitan Melbourne, while HeLP-her rural was set across 42 Victorian towns with a population of 2000 to 10,000, located between 100 and 400 kilometers from the Melbourne central business district [7,10]. This definition of rurality is in line with the Rural Remote and Metropolitan Areas classification [29]. All subjects gave their informed consent for inclusion before they participated in the study [7,10,28]. The study was conducted in accordance with the Declaration of Helsinki. HeLP-her urban protocol was approved by the Southern Health Human Research Ethics Committee (reference 05187C). HeLP-her rural protocol was approved by the Monash Health Human Research Ethics Committee (project number 12034B).

### 2.2. Participants

Participants included 250 mothers of primary school aged children recruited as part of HeLP-her urban from May 2006 to August 2006 [6] and 649 women recruited for HeLP-her rural from September 2012 to April 2013 [8]. Women across both RCTs were aged between 18–50 years of any weight [6,7,8,9,10], and resided in local communities of moderate socio-economic disadvantage based on the Socio-Economic Index for Areas [30]. Specific exclusion criteria included conditions or medications known to affect weight including prescribed weight control medication, bariatric surgery, and breastfeeding of infants under six months, pregnancy or a serious physical or psychological illness preventing complete study participation [6,7,8,9,10].

### 2.3. Baseline Measures

The current study analyzed baseline data for HeLP-her urban and HeLP-her rural participants. Pre-specified demographic, health, anthropometrics and medical history details were collected. All questionnaires and measures were administered by study staff.

### 2.4. Anthropometrics

All anthropometric measurements were performed by trained researchers [6,7,8,9,10]. Weight was measured on an electronic scale to the nearest 0.1 kg (Tanita WB110AZ) calibrated prior to weighing periods [6,7,8,9,10]. The weight of each participant was measured in light clothing without shoes and with an empty bladder [6,7,8,9,10]. Each participant’s height was measured using a portable stadiometer to the nearest 0.1 cm (Mentone Education Centre, Melbourne, Australia). BMI was calculated by dividing each participant’s weight in kilograms by height in meters squared [6,7,8,9,10]. The World Health Organization BMI classification system was used to classify participants as normal weight, overweight and obese [3].

### 2.5. Dietary Intake

Dietary intake was measured using a self-administered, semi-quantitative food frequency questionnaire (FFQ) from the Cancer Council of Victoria, for Epidemiological Studies Version 2 (DQESv2) [31]. The FFQ covers five types of core food groups including cereal foods, dairy products, meats and fish, fruit and vegetables and discretionary foods and beverages including sweets or savory snacks and alcoholic beverages, incorporating 80 food items [31]. Participants were asked to answer questions regarding their usual dietary intake over the last 12 months and indicate how frequently they consumed these foods from never, monthly, weekly, daily, up to three or more times per day [31]. The FFQ also contained pictures of meals for participants to indicate their usual portion size which was also included in the final dietary analysis. Dietary intake data were collected and analyzed using the Food Standards Australian New Zealand Nutrient Tables (NUTTAB 95) for use in Australia [32].

### 2.6. Dietary Quality

Diet quality was measured using a modified version of the Dietary Guideline Index (DGI) [33]. The DGI was updated to include the current Australian Dietary Guidelines [34] and recommendations from the Australian Guide to Healthy Eating (AGHE) [35]. Due to the FFQ not providing specific information to fulfill the requirements of salt use, fluid intake, and one of the saturated fat components that specified the trimming of fat from meat, these components were excluded [33]. Each component is scored from zero to ten, where zero indicates non adherence to the dietary guideline and ten indicates meeting the recommended dietary guideline [33]. The revised tool consisted of 13 items including dietary variety, vegetables, fruit, wholegrain cereals, breads and cereals, meat and meat alternatives, lean protein sources, dairy, the type of milk usually consumed and extra foods including alcohol and added sugars so that the total diet score had a range of 0–130 (Table 1). A higher score is indicative of greater compliance with the Australian Dietary Guidelines and therefore a higher diet quality [33].

### 2.7. Data Analysis

Consideration of plausible energy reporting strengthens the measured association between diet and health outcomes and reduces variability [36]. A common approach used to identify under and over-reporters in surveys is to compare each person’s basal metabolic rate (BMR) based on their age, sex and weight with their reported energy intake (EI) and apply Goldberg cut-off values to identify those with a plausible energy intake [37]. The EI/BMR (total energy intake over BMR) provides an indication of whether the reported energy intake is consistent with the energy intake required for a person to live a normal lifestyle [37]. The BMR for each women was calculated using the Schofield equation [38]. Based on prior recommendations, the Goldberg cut off of <0.9 for low energy reporters, 0.9–2.1 for adequate energy reporters and >2.1 for high energy reporters was applied [37,39]. Participants identified as low energy reporters and high energy reporters were excluded from the analysis. Statistical analyses were conducted with Stata Statistical Software: Release 12 (StataCorp LLC, College Station, TX, USA) [40], with a two-sided significance level. A statistical analysis plan was followed under the guidance of an experienced biostatistician. As outcome variables were normally distributed, all results were reported as mean ± standard deviation (SD), unless otherwise stated. Baseline characteristics for urban and rural women were compared using *t*-tests for continuous data and chi square tests for categorical data. Based on normal distributions in outcome variables, differences in energy, macronutrient and micronutrient intake and in diet quality total score and components in urban and rural women were analyzed using linear regression analysis for continuous outcomes. Logistic regression analysis were used for dichotomous outcomes (type of milk usually consumed) and generalized linear models using both linear and logistic regression were used for proportional outcomes (lean protein sources and wholegrain cereals). The variables in the adjusted model were based on a priori hypothesis testing, as each of these socio-demographic variables were clinically relevant to diet quality and were required to accurately interpret the results. As this is a cluster randomized controlled trial where women were recruited by town we adjusted for this accordingly, as per our previous papers [6,8].

## 3. Results

### 3.1. Participants

**Figure 1:**
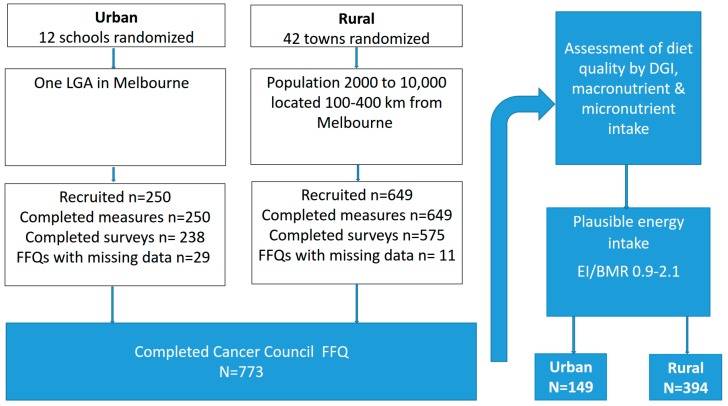
Flow chart of participants in the final analysis.

A total of 12 schools and 42 towns were randomized in the urban and rural cohorts, respectively. An initial 250 urban women were recruited and completed anthropometric measures and 238 completed surveys. In the rural cohort, 649 women were recruited and completed anthropometric measures and 575 completed surveys. Across the two cohorts, a total of 773 women fully completed an FFQ where diet quality as measured by the DGI and macronutrient and micronutrient intake was assessed. To minimize bias in dietary data reporting, only those with a plausible energy intake (EI/BMR 0.9–2.1) were included in final analysis. Using the Goldberg equation, 230 women were identified as misreporters (under reporters urban *n* = 53, rural *n* = 144; over reporters urban *n* = 7, rural *n* = 26), resulting in a total sample size of 149 urban and 394 rural women (Figure 1).

### 3.2. Participant Characteristics

The demographic characteristics of all participants are reported in Table 2. There were no significant differences in age, BMI, marital status and income between urban and rural women, however, significantly more rural women reported a higher educational attainment in comparison to urban women.

### 3.3. Macronutrient and Micronutrient Intake

Table 3 outlines the energy, macronutrient and micronutrient intake for all participants. After adjustment for qualification, income, working, BMI, age, marital status and town clustering, rural women had a higher intake in protein, fat (g and % energy), saturated and monounsaturated fats (g and % energy), cholesterol and iron and a lower intake of polyunsaturated fat (% energy) compared to urban women. There were no significant differences between urban and rural women for total energy, carbohydrate, fiber, glycemic index(GI), glycemic load(GL), calcium, folate and sodium on adjusted analyses.

### 3.4. Diet Quality

There were no differences in the total DGI for urban and rural women on unadjusted or adjusted analysis (Table 4). Rural women had a significant higher score in the diet quality component relating to meat and meat alternatives component in comparison to urban women in both the unadjusted and adjusted analysis. All other components of the DGI were not significantly different between urban and rural women on unadjusted or adjusted analysis.

### 3.5. Predictors of Diet Quality in All Women

Demographic variables that were significantly associated with diet quality on unadjusted analysis were higher age, working status, a higher educational attainment, and a higher annual income (Table 5). On adjusted analysis working status and having a higher annual income remained associated with a higher diet quality (Table 5). Compared to working women, those that were not working had a significantly lower diet quality (−4.1, 95% CI −8.1 to −0.14 *p* = 0.043) and women on a higher income ($AUD80,000 and above per year) had a higher diet quality (5.5, 95% CI 1.2 to 9.8 *p* = 0.013) compared to women on a lower income (≤$AUD40,000 per year).

## 4. Discussion

Here, we advance the literature by comparing diet quality and nutrient intake between urban and rural Australian women of reproductive-age. Overall, we report no significant difference in diet quality between our cohorts. We evaluated predictors of diet quality and report that higher income levels and working status are associated with better diet quality in all women of reproductive age.

Our results differ from the majority of previous studies reporting higher diet quality generally in urban, compared to rural women [18,41,42,43]. However, two previous Australian studies in mid aged women aged 50–55 years from the Australian Longitudinal Study of Women’s Health reported no difference in diet quality between urban and rural locality [18,24]. These studies assessed diet quality using a different diet quality index, the Australian Recommended Food Score (ARFS) developed in accordance with the AGHE and validated in mid-aged Australian women [44]. While both the ARFS and DGI reflect adherence to the AGHE, there are slight variations in scoring methods, assignment of items to food groups and cut off values [18]. It is possible that the DGI is more sensitive to the particular dietary differences that we observed here. It is also possible that slight differences in the food preferences between urban and rural populations exist, but that these differences may not have an effect on overall diet quality.

We observed here that income levels and working status significantly predicted better diet quality overall. Previous reports demonstrate a higher educational attainment [45], income [45], and full or part time employment [46] are associated with a higher diet quality in women, and those who are not working have a significantly lower diet quality, consistent with our results. These results emphasize the need for effective healthier eating strategies for women and their families who are unemployed and on a lower annual income. Previous research demonstrates higher costs associated with eating a healthful diet [47,48] with the average cost for a healthy food basket for a typical family representing over a third (31%) of families’ household income [14]. Women from low income families often make sacrifices to their diet by reducing food quality and quantity of healthy foods purchased, and providing healthier foods/meals for their partner or children before their own [49]. These strategies are adopted in order to stretch out the budget and manage other basic needs including shelter, fuel, clothing and other essential commodities [49]. A recent systematic review, which aimed to determine the impact of food subsidy programs on the nutritional intake and health status of disadvantaged adults in high income countries, reported an increase in fruit and vegetable intake by 1–2 serves per day in disadvantaged women through targeted fruit and vegetable subsidies with nutrition education [50]. However, due to the small number of studies (*n* = 9) and the moderate to high risk of bias, further rigorous studies investigating the impact of food subsidy programs on participants nutritional intake and health outcomes is required [50]. Since this review, successful strategies such as healthy food vouchers [51] and supplemental food packages [52], designed to assist low income women and their children to improve their nutrition, have proven to be effective in these disadvantaged populations and therefore could be considered in the local context given the prevalence of obesity [2], longitudinal weight gain [1,53] and obesity associated chronic diseases [54] among women.

We also report a greater proportion of rural women with a university degree or higher compared to urban women. This is in contrast to national reports indicating a lower educational attainment in rural compared to urban areas [55]. The higher educational attainment in rural women reported here may contribute to a higher diet quality in our study population, potentially translating to reducing previously observed differences between urban and rural cohorts.

We report higher intakes of protein, total fat, saturated fatty acid (SFA), monounsaturated fatty acid (MUFA), iron and cholesterol in rural compared to urban women, although we note these differences were relatively small and therefore may not be clinically relevant. A similar dietary pattern of a higher protein, fat and cholesterol intake has been reported in Australian women living in remote areas in comparison to women living in inner rural and urban areas [56]. In our study, this dietary profile was also observed in association with a higher score in the diet quality component related to higher intake of lean meats or alternatives. This diet quality component consists of a variety of foods including lean meats (beef, chicken, lamb, pork, veal, and fish), legumes, nuts, tofu and eggs. We are unable to specify which of these foods are consumed in higher quantities in rural women. Rural women may have a greater contact with farm animals in comparison to urban women due to geographical location and occupation and this may contribute to a higher intake of meat products. Previous research suggests women in regular contact with farm animals have a more relaxed attitude to farm production and consumption of animal meals in comparison to females with no regular contact with animal farms [57]. Future research may benefit from further exploration of the cultural significance of meat consumption between urban and rural women.

The strengths of this study include the use of minimal exclusion criteria to optimize generalizability and equal representation of women across all BMI categories. We acknowledge several limitations of the present study. Data collected from the two different comparison cohorts were six years apart. It is therefore possible that food availability and eating patterns may have changed during this period, and therefore may be an invalid comparison. There are differences in recruitment methods between cohorts as the urban women were recruited from schools and the rural women were recruited from towns. The educational attainment of the rural women was significantly higher than the urban women, which creates the possibility of bias in the results. Limitations of self-report FFQs include misreporting, recall error, measurement error and social desirability bias [58]. Applying the Goldberg cut off to exclude women with an implausible energy intake resulted in a loss of 29.76% of participants, of which 25.49% were under reporters and 4.27% were over reporters. The percentage of women classified as under reporters is consistent with the percentage of female under reporters aged 31–50 years identified from the 2011–2012 National Nutrition and Physical activity survey [37]. The FFQ in the present study was developed and validated against weighed food records with Australian populations, and has been shown to provide a useful method of measuring habitual dietary intake in population settings [59,60]. Due to difficulty in matching demographic variables between urban and rural women from the HeLP-her baseline RCTs, we were unable to investigate parity [61], smoking status [33], and physical activity levels [33], all of which are known to influence diet quality in women. The association of these factors with diet quality in urban and rural women should be considered in future studies.

## 5. Conclusions

Our results show for the first time that there was no difference in total diet quality between urban and rural women of reproductive-age, but subtle differences in the macronutrient consumption patterns, potentially related to a higher lean meat intake in rural women. These findings highlight the association of diet quality with working status and income level, emphasizing the need to provide additional support and strategies to disadvantaged women and their families that facilitate healthier eating that is cost effective to improve overall diet quality. This is crucial to assist in the prevention of weight gain and obesity, which not only has negative consequences for reproductive health, pregnancy and birthing outcomes, but can also lead to adverse health in their older years. These findings may help to inform the development of targeted programs to improve nutrition in women to prevent weight gain and reduce risk for ill health and chronic disease.

## Figures and Tables

**Table 1:** Modified version of the Dietary Guideline Index (DGI).

2013 Australian Dietary Guidelines	DGI Component and Description	Maximum Score (10)	Intermediate Score (5)	No (0)
Enjoy a wide variety of nutritious foods	Dietary variety: proportion of foods for each core food group that were consumed at least once per week	100%	50%	0%
Eat plenty of vegetables, legumes and fruits	Vegetables: servings of vegetables and legumes per day	≥5	2.5	0
	Fruit: servings of fruit per day	≥2	1	0
Eat plenty of cereals (including breads, rice, pasta and noodles), preferably wholegrain	Breads and cereals: frequency of consumption of breads and cereals per day	≥6	3	0
	Wholegrain cereals: proportion of whole meal/wholegrain bread consumed relative to total bread	100%	50%	0%
Include lean meat, fish, poultry or alternatives	Meat and meat alternatives: frequency of consumption of lean meats and alternatives per day	≥2.5	1.25	0
	Lean protein sources: proportion of lean meats & alternatives relative to total meats and alternatives	100%	50%	0%
Include milks, yoghurts, cheeses and/or alternatives Reduced fat varieties should be chosen, where possible	Dairy: frequency of consumption of dairy products per day	≥2.5	1.25	0
	Saturated fat intake: type of milk usually consumed	Low fat milk		Whole milk
Limit saturated fat intake and moderate total fat intake	Saturated fat intake: type of milk usually consumed	Low fat milk		Whole milk
Limit your alcohol intake if you choose to drink	Alcohol: frequency of consumption of all alcoholic beverages per day	≤1	1.5	≥2
Consume only moderate amounts of sugars and foods containing added sugars	Added sugars: frequency of consumption of soft drink, cordial, fruit juice, jam, chocolate, confectionary per day	<1.25	1.25	>1.25
Prevent weight gain: by being physically active and eating according to your energy needs	Extra foods: frequency of consumption of extra foods per day	<2.5	2.5	>2.5
	TOTAL DGI SCORE	0–130		

**Table 2:** Baseline demographic characteristics of urban and rural women.

Variables	Urban (*n* = 149)	Rural (*n* = 394)	*p*-Value
Age (years)	40.4 ± 4.4	39.7 ± 6.4	0.227
BMI (kg/m^2^)	27.6 ± 5.6	27.7 ± 6.0	0.860
Employment			
Working	96 (64.9%)	291 (74.8%)	0.093
Not working	52 (35.1%)	98 (25.2%)	
Marital status			
Never married	4 (2.7%)	24 (6.1%)	0.306
Married	131 (87.9%)	338 (86.2%)	
No longer married	14 (9.4%)	30 (7.7%)	
Education			
No formal	67 (45.0%)	60 (15.4%)	<0.001
Trade/apprentice ^a^	39 (26.2%)	185 (47.3%)	
University degree or higher	43(28.9%)	146 (37.3%)	
Income			
≤$AUD40,000	28 (21.7%)	77 (20.6%)	0.792
$AUD41–80,000	60 (46.5%)	164 (43.9%)	
$AUD80,000 and above	41 (31.8%)	133 (35.6%)	

Data are presented as mean ± SD or frequency and percentage and were analyzed by *t*-test for continuous data and chi square test for categorical data adjusted for town clustering. ^a^ Includes trade, apprenticeship, certificate and diploma. Abbreviation: BMI—body mass index, $AUD—Australian dollar.

**Table 3:** Energy, macronutrient and micronutrient intake for urban and rural women.

Nutrients	Urban (*n* = 149)	Rural (*n* = 394)	Unadjusted β (95% Confidence Interval) (CI)	*p*-Value	Adjusted ^a^ β (95% CI)	*p*-Value
Energy (kJ/day)	7644.3 ± 1905.9	7965.4 ± 1930.5	321.1 (−38.7, 680.9)	0.079	360.8 (−42.4, 764.1)	0.078
Protein (g/day)	87.2 ± 26.2	93.7 ± 28.6	6.5 (2.2, 10.8)	0.004	7.0 (1.7, 12.3)	0.010
% Protein	19.3	20	0.007 (0.0002, 0.01)	0.044	0.007 (−0.0006, 0.01)	0.073
CHO (g/day)	188.1 ± 52.0	189.1 ± 51.1	1.0 (−10.1, 12.1)	0.857	2.5 (−9.2, 14.2)	0.668
% CHO	39.4	38	−0.01(−0.03, −0.001)	0.031	−0.01 (−0.03, 0.0005)	0.059
Fat (g/day)	73.5 ± 23.7	79.3 ± 23.3	5.8 (0.79, 10.7)	0.024	6.7 (1.6, 11.8)	0.011
% Fat	35.3	36.6	0.01 (0.002, 0.02)	0.026	0.02 (0.006, 0.03)	0.004
SFA (g/day)	29.3 ± 11.0	32.9 ± 11.3	3.6 (1.0, 6.1)	0.007	3.9 (1.4, 6.3)	0.003
% SFA	14	15.1	0.01 (0.004, 0.02)	0.003	0.01 (0.006, 0.02)	<0.001
MUFA (g/day)	26.3 ± 9.1	28.5 ± 8.6	2.2 (0.49, 3.9)	0.012	2.6 (0.81, 4.3)	0.005
% MUFA	12.6	13.1	0.005 (0.0008, 0.01)	0.022	0.007 (0.002, 0.01)	0.005
PUFA (g/day)	11.4 ± 4.6	11.0 ± 4.1	−0.40 (−1.1, 0.34)	0.282	−0.17 (−0.96, 0.62)	0.670
% PUFA	5.5	5.1	−0.004 (−0.006, −0.001)	0.003	−0.003 (−0.005, −0.0004)	0.023
Fibre (g/day)	21.3 ± 7.0	21.6 ± 6.1	0.34 (−0.95, 1.6)	0.600	0.46 (−0.77, 1.7)	0.459
Cholesterol (mg/day)	267.0 ± 106.7	314.6 ± 112.4	47.6 (24.7, 70.6)	<0.001	49.4 (25.4, 73.4)	<0.001
GI	52.2 ± 3.6	50.9 ± 4.0	−1.3 (−2.0, −0.64)	<0.001	−0.76 (−1.6, 0.04)	0.062
GL	97.8 ± 29.5	96.1 ± 29.9	−1.7 (−8.4, 4.9)	0.601	0.11 (−6.9, 7.1)	0.974
Calcium (mg/day)	897.7 ± 272.5	925.4 ± 273.0	27.7 (−15.7, 71.0)	0.207	4.0 (−38.6, 46.6)	0.850
Iron (mg/day)	12.6 ± 4.0	13.6 ± 4.0	0.97 (0.43, 1.5)	0.001	1.1 (0.48, 1.6)	0.001
Folate (µg/day)	257.1 ± 80.4	267.1 ± 79.8	10.0 (−1.8, 21.8)	0.094	9.6 (−2.3, 21.5)	0.113
Sodium (mg/day)	2517.5 ± 779.5	2525.0 ± 756.9	7.5 (−138.6, 153.6)	0.918	30.5 (−142.5, 203.5)	0.725

Data are presented as mean ± SD and β (95% CI) and were analyzed using linear regression analysis. ^a^ Adjusted for education, income, working, body mass index, age, marital status and town clustering. Abbreviations: CHO—carbohydrate, SFA—saturated fat, MUFA—monounsaturated fat, PUFA—polyunsaturated fat, GI—glycemic index, GL—glycemic load.

**Table 4:** Difference in diet quality components between urban and rural women.

DGI and Components	Urban (*n* = 149) DGI Score	Rural (*n* = 394) DGI Score	Unadjusted β (95% Confidence Interval) (CI)	*p*-Value	Adjusted ^a^ β (95% Confidence Interval) (CI)	*p*-Value
Dietary variety	0.66 ± 0.08	0.65 ± 0.10	−0.01 (−0.03, 0.007)	0.206	−0.02 (−0.04, 0.001)	0.066
Vegetables	2.2 ± 0.96	2.4 ± 1.0	0.15 (−0.01, 0.32)	0.073	0.15 (−0.07, 0.36)	0.174
Fruit	1.6 ± 0.97	1.6 ± 1.0	−0.03 (−0.20, 0.15)	0.770	0.0004 (−0.17, 0.18)	0.996
Wholegrain cereals	0.68 ± 0.46	0.69 ± 0.46	0.04 (−0.36, 0.44)	0.832	−0.11 (−0.58, 0.36)	0.636
Breads and cereals	4.4 ± 1.6	4.2 ± 1.6	−0.25 (−0.54, 0.04)	0.086	−0.18 (−0.55, 0.19)	0.336
Meat and meat alternatives	2.1 ± 1.2	2.4 ± 1.3	0.33 (0.12, 0.53)	0.002	0.37 (0.14, 0.61)	0.003
Lean protein sources	0.83 ± 0.12	0.82 ± 0.10	−0.03 (−0.17, 0.11)	0.668	0.004 (−0.14, 0.14)	0.952
Dairy	1.7 ± 0.72	1.8 ± 0.72	0.10 (−0.03, 0.23)	0.128	0.04 (−0.08, 0.16)	0.546
Low fat/skim milk whole milk (frequency & percentage) (%)						
Whole milk	54 (36.2%)	170 (43.2%)				
Low fat/skim milk	95 (63.8%)	224 (56.9%)	0.75 (0.48, 1.2)	0.193	0.63 (0.38, 1.1)	0.081
Saturated fat Low fat/skim milk whole milk (frequency & percentage) (%)						
Whole milk	54 (36.2%)	170 (43.2%)				
Low fat/skim milk	95 (63.8%)	224 (56.9%)	0.75 (0.48, 1.2)	0.193	0.63 (0.38, 1.1)	0.081
Extra foods ^b^	4.4 ± 1.9	4.6 ± 2.2	0.12 (−0.34, 0.57)	0.612	0.08 (−0.38, 0.54)	0.727
DGI total	84.8 ± 15.9	83.9 ± 16.5	−0.90 (−4.4, 2.6)	0.606	−1.8 (−5.1, 1.4)	0.264

Data are presented as mean ± SD and β (95% CI) and were analyzed by linear regression analysis. Proportional variables (wholegrain cereals and lean protein sources) were analyzed by generalized linear models. Low fat/skim milk whole milk and saturated fat components were analyzed by logistic regression. ^a^ Adjusted for education, income, working, body mass index, age, marital status and town clustering; ^b^ Includes alcohol and added sugars components.

**Table 5:** Contributors of baseline demographic and anthropometric factors to total diet quality for urban and rural women.

Variables	Unadjusted β (95% CI)	*p*-Value	Adjusted ^b^ β (95% CI)	*p*-Value
Rural status	−0.90 (−4.4, 2.6)	0.606	−1.8 (−5.1, 1.4)	0.264
Age (years)	0.26 (0.06, 0.46)	0.012	0.25 (−0.02, 0.52)	0.068
BMI (kg/m^2^)	0.03 (−0.21, 0.26)	0.805	0.12 (−0.12, 0.36)	0.324
Employment				
Working	Ref (1)			
Not working	−5.6 (−9.1, −2.0)	0.003	−4.1 (−8.1, −0.14)	0.043
Marital status				
Married	Ref (1)			
Never married	3.0 (−3.6, 9.7)	0.367	1.8 (−5.8, 9.3)	0.639
No longer married	−0.71 (−8.9, 7.5)	0.862	−3.0 (−7.9, 1.9)	0.225
Education				
No formal	Ref (1)			
Trade/apprentice ^a^	0.61 (−2.6, 3.8)	0.703	0.82 (−3.7, 5.4)	0.720
University degree and higher	4.1 (0.90, 7.3)	0.013	3.3 (−0.94, 7.6)	0.124
Income				
$≤AUD40,000	Ref (1)			
$AUD41–80,000	3.8 (0.46, 7.1)	0.026	2.6 (−1.2, 6.5)	0.176
$AUD80,000 and above	7.6 (3.6, 11.6)	<0.001	5.5 (1.2, 9.8)	0.013

Data are presented as β (95% CI) *p*-value and were analyzed by linear regression analysis. ^a^ Includes trade, apprenticeship, certificate and diploma; ^b^ Adjusted for education, income, working, body mass index, age, marital status and town clustering. Abbreviation: BMI—body mass index, $AUD—Australian dollar.

## Abbreviations

The following abbreviations are used in this manuscript:

$AUD	Australian dollar
DGI	Dietary Guideline Index
BMI	Body Mass Index
HeLP-her	The Healthy Lifestyle Program
FFQ	Food Frequency Questionnaire
EI/BMR	Energy Intake: Basal Metabolic Rate
AGHE	Australian Guide to Healthy Eating
SD	Standard Deviation
N	Number
ARFS	Australian Recommended Food Score
CHO	Carbohydrate
SFA	Saturated fatty acid
MUFA	Monounsaturated fatty acid
PUFA	Polyunsaturated fatty acid
GI	Glycemic Index
GL	Glycemic load

## References

[1] Brown W. Australian women and their weight: A growing problem. Proceedings of the Meeting of Commonwealth Department of Health and Ageing.

[2] World Health Organization Obesity and Overweight. http://www.who.int/mediacentre/factsheets/fs311/en/.

[3] World Health Organization (2000). Obesity: Preventing and Managing the Global Epidemic.

[4] Zugravu C.A. (2012). Eating habits and influential factors for mothers and children in Romania. Int. J. Collab. Res. Intern. Med. Public Health.

[5] Smith K.J., McNaughton S.A., Gall S.L., Blizzard L., Dwyer T., Venn A.J. (2010). Involvement of young Australian adults in meal preparation: Cross-sectional associations with sociodemographic factors and diet quality. J. Am. Diet. Assoc..

[6] Smith K.B., Humphreys J.S., Wilson M.G. (2008). Addressing the health disadvantage of rural populations: How does epidemiological evidence inform rural health policies and research. Aust. J. Rural Health.

[7] Eberhardt M.S., Pamuk E.R. (2004). The importance of place of residence: Examining health in rural and nonrural areas. Am. J. Public Health.

[8] World Health Organization (2010). Rural Poverty and Health Systems in the WHO European Region.

[9] Palermo C., McCartan J., Kleve S., Sinha K., Shiell A. (2016). A longitudinal study of the cost of food in Victoria influenced by geography and nutritional quality. Aust. N. Z. J. Public Health.

[10] World Health Organization Women’s Health Fact Sheet Number 334. http://www.who.int/mediacentre/factsheets/fs334/en/.

[11] Wirt A., Collins C.E. (2009). Diet quality-what is it and does it matter. Public Health Nutr..

[12] Schwingshackl L., Hoffmann G. (2015). Diet quality as assessed by the healthy eating index, the alternate healthy eating index, the dietary approaches to stop hypertension score, and health outcomes: A systematic review and meta-analysis of cohort studies. J. Acad. Nutr. Diet..

[13] Alhazmi A., Stojanovski E., McEvoy M., Brown W., Garg M.L. (2014). Diet quality score is a predictor of type 2 diabetes risk in women: The Australian longitudinal study on women’s health. Br. J. Nutr..

[14] Huot I., Paradis G., Receveur O., Ledoux M. (2004). Correlates of diet quality in the Quebec population. Public Health Nutr..

[15] Pedroza-Tobías A., Hernández-Barrera L., López-Olmedo N., García-Guerra A., Rodríguez-Ramírez S., Ramírez-Silva I., Villalpando S., Carriquiry A., Rivera J.A. (2016). Usual vitamin intakes by Mexican populations. J. Nutr..

[16] Bojar I., Owoc A., Humeniuk E., Fronczak A., Walecka I. (2014). Quality of pregnant women’s diet in Poland-macro-elements. Arch. Med. Sci..

[17] Ntandou G., Delisle H., Agueh V., Fayomi B. (2009). Abdominal obesity explains the positive rural-urban gradient in the prevalence of the metabolic syndrome in Benin, West Africa. Nutr. Res..

[18] Tsigga M., Filis V., Hatzopoulou K., Kotzamanidis C., Grammatikopoulou M.G. (2011). Healthy eating index during pregnancy according to pre-gravid and gravid weight status. Public Health Nutr..

[19] Potter J.L., Collins C.E., Brown L.J., Hure A.J. (2014). Diet quality of Australian breast cancer survivors: A cross-sectional analysis from the Australian longitudinal study on women’s health. J. Hum. Nutr. Diet..

[20] Aljadani H.M., Patterson A., Sibbritt D., Hutchesson M.J., Jensen M.E., Collins C.E. (2013). Diet quality, measured by fruit and vegetable intake, predicts weight change in young women. J. Obes..

[21] Tobias D., Zhang C., Chavarro J., Olsen S., Bao W., Bjerregaard A., Fung T., Manson J., Hu F. (2016). Healthful dietary patterns and long-term weight change among women with a history of gestational diabetes mellitus. Int. J. Obes..

[22] Zamora D., Gordon-Larsen P., He K., Jacobs D.R., Shikany J.M., Popkin B.M. (2011). Are the 2005 dietary guidelines for Americans associated with reduced risk of type 2 diabetes and cardiometabolic risk factors? Twenty-year findings from the cardia study. Diabetes Care.

[23] Lombard C., Deeks A., Jolley D., Teede H.J. (2009). Preventing weight gain: The baseline weight related behaviors and delivery of a randomized controlled intervention in community based women. BMC Public Health.

[24] Lombard C.B., Harrison C.L., Kozica S.L., Zoungas S., Keating C., Teede H.J. (2014). Effectiveness and implementation of an obesity prevention intervention: The HeLP-her rural cluster randomised controlled trial. BMC Public Health.

[25] Harrison C., Teede H., Kozica S., Zoungas S., Lombard C. (2016). Individual, social and environmental factors and their association with weight in rural dwelling women of reproductive age. Aust. N. Z. J. Public Health.

[26] Lombard C., Deeks A., Jolley D., Ball K., Teede H. (2010). A low intensity, community based lifestyle programme to prevent weight gain in women with young children: Cluster randomized controlled trial. BMJ.

[27] Lombard C., Harrison C., Kozica S., Zoungas S., Ranasinha S., Teede H. (2016). Preventing weight gain in women in rural communities: A cluster randomised controlled trial. PLoS Med..

[28] Australian Institute of Health and Welfare (2004). Rural, Regional and Remote Health: A Guide to Remoteness Classifications.

[29] Australian Bureau of Statistics (2011). 2033.0.55.001-Census of Population and Housing: Socio-Economic Indexes for Areas (SEIFA), Australia. http://www.abs.gov.au/websitedbs/censushome.nsf/home/seifa2011?opendocument&navpos=260.

[30] Lombard C.B., Deeks A.A., Ball K., Jolley D., Teede H.J. (2009). Weight, physical activity and dietary behavior change in young mothers: Short term results of the help-her cluster randomized controlled trial. Nutr. J..

[31] Cancer Council Victoria Dietary Questionnaire for Epidemiological Studies Version 2. http://www.cancervic.org.au/research/epidemiology/nutritional_assessment_services.

[32] Lewis J., Milligan G.C., Hunt A. (1995). Nuttab 95: Nutrient Data Table for Use in Australia.

[33] McNaughton S.A., Ball K., Crawford D., Mishra G.D. (2008). An index of diet and eating patterns is a valid measure of diet quality in an Australian population. J. Nutr..

[34] National Health and Medical Research Council (2013). Australian Dietary Guidelines.

[35] National Health and Medical Research Council Australian Guide to Healthy Eating. https://www.eatforhealth.gov.au/guidelines/australian-guide-healthy-eating.

[36] Jessri M., Lou W.Y., L’Abbé M.R. (2016). Evaluation of different methods to handle misreporting in obesity research: Evidence from the Canadian national nutrition survey. Br. J. Nutr..

[37] Australian Bureau of Statistics 4363.0.55.001—Australian Health Survey: Users’ Guide, 2011–13 Under-Reporting in Nutrition Surveys. http://www.abs.gov.au/ausstats/abs@.nsf/Lookup/4363.0.55.001Chapter651512011-13.

[38] Schofield W. (1984). Predicting basal metabolic rate, new standards and review of previous work. Hum. Nutr. Clin. Nutr..

[39] Black A.E. (2000). Critical evaluation of energy intake using the Goldberg cut-off for energy intake: Basal metabolic rate. A practical guide to its calculation, use and limitations. Int. J. Obes..

[40] (2011). Stata Statistical Software: Release 12.

[41] Lutfiyya M.N., Chang L.F., Lipsky M.S. (2012). A cross-sectional study of US rural adults’ consumption of fruits and vegetables: Do they consume at least five servings daily. BMC Public Health.

[42] Suliga E. (2015). Nutritional behaviours of pregnant women in rural and urban environments. Ann. Agric. Environ. Med..

[43] Trivedi T., Liu J., Probst J., Merchant A., Jhones S., Martin A. (2015). Obesity and obesity-related behaviors among rural and urban adults in the USA. Rural. Remote. Health.

[44] Collins C.E., Young A.F., Hodge A. (2008). Diet quality is associated with higher nutrient intake and self-rated health in mid-aged women. J. Am. Coll. Nutr..

[45] Kant A.K. (2004). Dietary patterns and health outcomes. J. Am. Diet. Assoc..

[46] Miller J., Chan L., Mehta K., Roberts R., Dickinson K.M., Yaxley A., Matwiejczyk L., Thomas J., Wray A., Jackson K. (2016). Dietary intake of working women with children does not appear to be influenced by hours of employment: A secondary analysis of the Australian health survey (2011–2013). Appetite.

[47] Lee J.H., Ralston R.A., Truby H. (2011). Influence of food cost on diet quality and risk factors for chronic disease: A systematic review. Nutr. Diet..

[48] Rao M., Afshin A., Singh G., Mozaffarian D. (2013). Do healthier foods and diet patterns cost more than less healthy options? A systematic review and meta-analysis. BMJ Open.

[49] Grant D.K., Maxwell S. (1999). Food coping strategies: A century on from rowntree. Nutr. Health.

[50] Black A.P., Brimblecombe J., Eyles H., Morris P., Vally H., O’Dea K. (2012). Food subsidy programs and the health and nutritional status of disadvantaged families in high income countries: A systematic review. BMC Public Health.

[51] McFadden A., Green J.M., Williams V., McLeish J., McCormick F., Fox-Rushby J., Renfrew M.J. (2014). Can food vouchers improve nutrition and reduce health inequalities in low-income mothers and young children: A multi-method evaluation of the experiences of beneficiaries and practitioners of the healthy start programme in England. BMC Public Health.

[52] Odoms-Young A.M., Kong A., Schiffer L.A., Porter S.J., Blumstein L., Bess S., Berbaum M.L., Fitzgibbon M.L. (2014). Evaluating the initial impact of the revised special supplemental nutrition program for women, infants, and children (WIC) food packages on dietary intake and home food availability in African-American and hispanic families. Public Health Nutr..

[53] Dennis B., Pajak A., Pardo B., Davis C., Williams O., Piotrowski W. (2000). Weight gain and its correlates in Poland between 1983 and 1993. Int. J. Obes. Relat. Metab. Disord..

[54] Hruby A., Manson J.E., Qi L., Malik V.S., Rimm E.B., Sun Q., Willett W.C., Hu F.B. (2016). Determinants and consequences of obesity. Am. J. Public Health.

[55] Australian Institute of Health and Welfare (2008). Rural, Regional and Remote Health: Indicators of Health Status and Determinants of Health Rural Health Series.

[56] Mishra G., Ball K., Patterson A., Brown W., Hodge A., Dobson A. (2005). Socio-demographic inequalities in the diets of mid-aged Australian women. Eur. J. Clin. Nutr..

[57] Kubberød E., Ueland Ø., Tronstad Å., Risvik E. (2002). Attitudes towards meat and meat-eating among adolescents in Norway: A qualitative study. Appetite.

[58] Dietary Assessment Primer Food Frequency Questionnaire at a Glance. http://dietassessmentprimer.cancer.gov/profiles/questionnaire/.

[59] Hodge A., Patterson A.J., Brown W.J., Ireland P., Giles G. (2000). The anti cancer council of Victoria FFQ: Relative validity of nutrient intakes compared with weighed food records in young to middle-aged women in a study of iron supplementation. Aust. N. Z. J. Public Health.

[60] Ireland P., Jolley D., Giles G., O’Dea K., Powles J., Rutishauser I., Wahlqvist M.L., Williams J. (1994). Development of the Melbourne FFQ: A food frequency questionnaire for use in an Australian prospective study involving an ethnically diverse cohort. Asia Pacific J. Clin. Nutr..

[61] Bodnar L.M., Siega-Riz A.M. (2002). A diet quality index for pregnancy detects variation in diet and differences by sociodemographic factors. Public Health Nutr..

